# Longitudinal evaluation of macular vascular density alterations in unilateral amblyopic children undergoing therapy: An optical coherence tomographic angiography study

**DOI:** 10.1016/j.heliyon.2024.e31899

**Published:** 2024-05-24

**Authors:** Gorka Sesma, Tasnim Almairi, Heba Khashoggi, Fahad Aljohar, Rajiv Khandekar, Abdulaziz Awad

**Affiliations:** a Pediatric Ophthalmology and Strabismus Division, King Khaled Eye Specialist Hospital, Riyadh, Riyadh, Saudi Arabia; b Emergency Department, Almoosa Specialist Hospital, Al Mubarraz, Saudi Arabia; c Diagnostic and Imaging Department, King Khaled Eye Specialist Hospital, Riyadh, Riyadh, Saudi Arabia; d Research Department, King Khaled Eye Specialist Hospital, Riyadh, Riyadh, Saudi Arabia; e Khandekar Research Consultancy Services, Montreal, Canada

**Keywords:** Amblyopia, Retinal vascular density, OCTA, Occlusion therapy, Vision recovery

## Abstract

**Importance:**

Understanding the pathophysiology of the macula in amblyopic eyes is an active research area.

**Objective:**

To correlate macular retinal vascular density changes with best-corrected visual acuity changes following occlusion therapy for amblyopia in children.

**Design:**

A prospective cohort study of children visiting the Pediatric Ophthalmology Division of our institution between January 2020 and January 2022 was conducted.

**Setting:**

A specialist eye hospital in Saudi Arabia.

**Participants:**

Thirty children with unilateral amblyopia.

**Exposure:**

Occlusion therapy for amblyopia.

Main Outcome and Measures: Best corrected visual acuity (logMAR) before and at each of the four optical coherence tomographic angiographies was compared in amblyopic and fellow eyes. The effect of pretreatment determinants on the correlation between best-corrected visual acuity and retinal vascular density changes was reviewed.

**Results:**

In this cohort of 30 amblyopic and 30 fellow eyes from 30 children (mean age 8.7 ± 1.4 years; male: female 18:12. The best-corrected visual acuity improved from a median of 0.6 (interquartile range 0.5; 1.1) pretreatment to a median of 0.4 (interquartile range 0.2; 0.6) posttreatment in amblyopic eyes, and from a median of 0.1 to 0.05 in the fellow eyes. The total percentage change in retinal vascular density in the amblyopic eye was significantly higher than that in the fellow eye (*Z* = −1.92, *P* = 0.05). The change in best-corrected visual acuity in the amblyopic eye after a median of 98 months (interquartile range, 69–126 months) of intervention was significantly correlated with the refraction-adjusted change in retinal vascular density (*B* = −0.03, 95 % confidence interval −0.04, −0.02, *P* < 0.001) and was influenced by strabismus (*B* = −0.46, 95 % confidence interval −0.59, −0.34, *P* < 0.001), type of amblyopia (*B* = 0.24, 95 % confidence interval 0.12, 0.36, *P* < 0.001), duration of occlusion (*B* = −0.43, 95 % confidence interval −0.65, −0.22, *P* < 0.001), and occlusion compliance (*B* = 0.24, 95 % confidence interval 0.11, 0.36, *P* < 0.001).

**Conclusions:**

and Relevance: The RVD in amblyopic eyes in the first six months of therapy was significantly lower than that in fellow eyes, but not in subsequent assessments.

## 1 Introduction

Optical coherence tomographic angiography (OCTA) is approved by the US Food and Drug Administration and is commercially available since September 2015 [1]. OCTA has become a non-invasive and widely accepted tool among ophthalmologists in recent years [2]. It helps in studying the vascular structures of the retina and choroid and understanding the pathophysiology of several ocular conditions such as glaucoma, diabetic retinopathy, age-related macular degeneration, choroidal neovascularization, and amblyopia [[3], [4], [5], [6]]. OCTA detects blood flow-induced changes in OCT-reflected signals, and quantification of OCTA assists in monitoring the impact of the management and progression of retinal diseases [7]. OCT systems, such as the spectral domain (SD) and swept source (SS), are faster in the acquisition of images and thus are good for children [8]. Amblyopia is the leading cause of unilateral visual impairment in children and timely intervention could prevent visual disabilities [9]. Understanding the pathophysiology of the macula in amblyopic eyes is an active area of research. OCTA was used for this purpose along with fundus photography and fluorescein angiography. Retinal vascular density (RVD) in the superficial and deep plexuses in the macular area and foveal avascular zone is the main index used to study changes in macular perfusion patterns in amblyopic eyes [10]. There was a significant reduction in RVD at the macula in amblyopic eyes compared to healthy eyes. However, the difference between a unilateral amblyopic eye and a fellow eye remains contentious. Wong et al. [11] noted that 30 amblyopic eyes of children had an RVD of 97 % ± 2 while 1045 non-amblyopic eyes had an RVD of 97 % ± 1.7 and the difference was not significant (*P* = 0.61). However, the refractive status of the eye was not considered when comparing the indices. Araki et al. in their study compared 15 anisometric amblyopic eyes of Japanese children with fellow eyes. Both superficial (*P* = 0.43) and deep vascular density (*P* = 0.55) were not statistically significant [12]. The software had a built-in adjustment based on the axial length of the eyes. Parameters such as superior, inferior, and total RVD, percentage change, and rate of change micrometers/year to monitor macular vascular changes are currently inbuilt as indices in the OCTA software [13]. These indices have been used by researchers to correlate changes in RVD and visual acuity in amblyopic eyes relative to the fellow eye. In a small series of 12 amblyopic eyes, Gunzenhauser et al. [14] noted that macular vascular density increased from 53 % to 56 % (*P* = 0.046) more than 6.5 years after treatment. In contrast, Nishikawa et al. [15] documented a significantly lower magnification-corrected RVD in 22 unilateral amblyopic eyes than in their fellow eyes. Foveal SCD (*P* = 0.006) and foveal DCP (*P* = 0.024) were lower in the amblyopic eyes. Liu et al. [16] noted an interesting finding. A comparison group of fellow eyes and normal healthy children studying RVD in the amblyopic eye makes a difference. The perfusion of the macula in both the amblyopic and fellow eyes decreased. Lal et al. [17] noted that refractive ametropia can affect OCTA image magnification and indices. Therefore, it would be interesting to study refractive error-adjusted RVD parameters while correlating changes in RVD and best-corrected visual acuity (BCVA) in amblyopic eyes relative to fellow eyes. The literature has a small number of cases of amblyopic eyes undergoing OCTA before and after amblyopia treatment to review the magnification-adjusted perfusion of the macula and its correlation with changes in visual acuity.

We present changes in refraction-adjusted RVD indices noted on OCTA and BCVA at different stages of amblyopia therapy and describe the factors that influence their correlation among Saudi children.

## 2 Methods

The ethics and research committee of King Khaled Eye Specialist Hospital approved this study (20139-P). Informed written consent was obtained from the parents of the participating children. The tenets of the Declaration of Helsinki strictly adhered to this research project. Children with unilateral amblyopia who visited the Pediatric Ophthalmology Division of our institution between January 2015 and January 2020 were included in this study. Patients with unilateral occlusion were approached to participate in the study. If parents refused to undergo OCTA, their children were excluded from the study.

This research project was a prospective cohort study. To calculate the sample size, we assumed that 60 % of children had a change in the RVD and BCVA at the last OCTA assessment compared with that noted during the first OCTA in the amblyopic eye; however, in the fellow eye, such changes were only observed in 15 % of children. To achieve a 95 % confidence interval and 90 % power in the cohort study with a 1:1 ratio of amblyopic eye: fellow eye, 27 children were recruited. To compensate for data loss, 10 % of the sample was added. Thus, the final sample comprised 30 amblyopic and 30 fellow eyes from 30 children. We used Open epi software to calculate the sample size for the cohort study [18].

BCVA was measured in each eye using an Early Treatment Diabetic Retinopathy Study (ETDRS) chart held at a 6-m distance. The Snellen's measurement notations were in feet. If the child was unable to correctly identify the symbols of the top line, we retested their vision at 3 m and 1.5 m from the chart. Visual acuity was then converted to logMAR values. Amblyopia was defined as BCVA between 20/40 and 20/200, and the eye did not have an organic cause for decreased vision. The BCVA in the fellow eye should be 20/40 or better and there should be a minimum 2-line BCVA difference between the two eyes. Refraction was documented manually using an autorefractor after cycloplegia. The spherical equivalent (SE) was calculated as spherical + (cylinder/2) diopters (D). Hyperopia was categorized according to the degree of refractive error, which was measured in (D), as mild hyperopia (refractive error of +2.00 D or less), moderate hyperopia (refractive error greater than +2.00 up to +5.00 D), and severe (high) hyperopia (refractive error greater than +5.00 D). Mild myopia was characterized as a refractive error ranging from −0.50 to −3.00 D, while moderate myopia was defined as a refractive error ranging from −3.25 to −6.00 D. High myopia was classified as a refractive error greater than −6.00 D.

The type of strabismus was noted using a handheld torchlight held at a 1-m distance from the face while performing a cover uncover test and was termed exotropia, esotropia, hyper, and hypotropia. The degree of strabismus was measured using a synoptophore (Haag Streit; Koeniz, Switzerland). If the SE of one eye was ±1D different from the fellow eye, we described the child as having anisometropia. In our study, "mild" amblyopia was classified as visual acuity of 20/30 to 20/40, "moderate" amblyopia as being <20/40 to 20/100, and "severe" amblyopia as < 20/100 [19].

The eligibility criteria for this study were designed to encompass a broad range of refractive errors. Patients with amblyopia resulting from strabismus, anisometropia, or mixed conditions, regardless of severity, and any degree of refractive error, measured as spherical equivalent, were eligible to participate if they could maintain proper eye fixation during the OCTA procedure. This approach ensured a diverse population and made the findings applicable to a wide range of clinical scenarios. The study also considered strabismus, which can be either esotropia or exotropia. However, sensory strabismus was excluded to prevent the potential risk of fixation instability, which is essential for capturing accurate OCTA images. These criteria were established to balance the need for inclusivity with high-quality image acquisition.

In this study, foveal vascular density was measured using Spectral-domain OCTA (REVO NX Spectral Domain OCT with Angiography OCT Software; Optopol Technology, Zaweircie, Poland). A macular scan of 3 × 3 mm was initiated, encompassing both amblyopic and fellow eyes, according to the protocol followed by previous researchers [12,14]. To ensure the acquisition of clear images, the patients were coached for optimal cooperation, and our skilled technician, trained by the OCTA company, effectively utilized the machine's advanced features. Inbuilt software was used to automatically reject a scan if artifacts affected the interpretation of scan outcomes. The successful combination of patient cooperation and technical expertise is crucial for capturing high-quality, artifact-free images suitable for detailed analysis. Only patients who were capable of adhering to fixation protocols were included in the study.

**Fig. 1:**
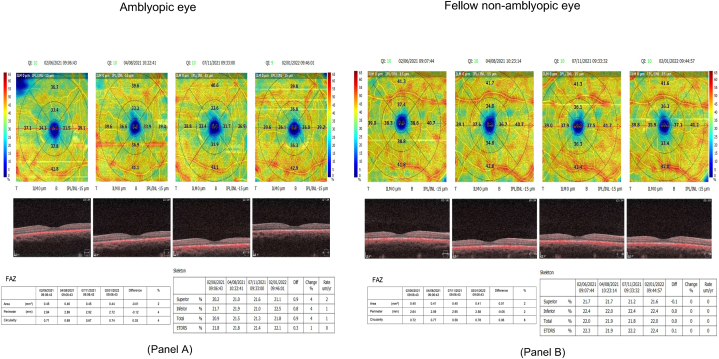
Retinal vascular density in amblyopic and fellow eyes. Panel A displays an Optical Coherence Tomography Angiography (OCTA) scan of the macula in an amblyopic eye. The image reveals reduced retinal vascular density (RVD) in comparison to the fellow eye. The OCTA scan shows thinner and fewer blood vessels in the macular region, which is indicative of impaired blood flow and structural anomalies commonly associated with amblyopia. The detailed view provided by the OCTA scan emphasizes areas of decreased perfusion, highlighting the vascular changes occurring in the amblyopic eye. Panel B displays an OCTA scan of the macula in the fellow non-amblyopic eye, which serves as a control. The image exhibits a normal distribution and density of retinal blood vessels, reflecting typical macular perfusion. The contrast between the vascular density in the amblyopic and non-amblyopic eyes is evident, emphasizing the vascular deficits present in the amblyopic eye.

The second, third, and fourth OCTAs of each eye were repeated at 3-month intervals. The indices generated by the software were documented for each OCTA image of the amblyopic and fellow eyes. The four OCTAs of the amblyopic eye and fellow eye along with the indices are given in Fig. 1. All patients received patching therapy as the primary treatment method. The adherence to patching was thoroughly assessed by the division's orthoptists. The duration of patching was individualized based on the severity of amblyopia, with 2 h recommended for mild to moderate cases and 6 h for severe cases, along with at least 1 h of near vision activities. The patients were monitored every three months, and this regimen was sustained for a period of one year.

The change in BCVA (logMAR) was defined as follows: BCVA at the start of the occlusion therapy and BCVA at the OCTA visit. Refraction-adjusted total RVD parameters were obtained at each OCTA visit.

Demographic and ocular data were collected from the computerized case records of the participants using a pretested form. This information was entered into a Microsoft Excel spreadsheet (Microsoft Corporation, Redmond, WA, USA). The data collected during the OCTA visits were transferred from the OCT printouts obtained from the Department of Digital Imaging. The information in the spreadsheet was organized into data of the amblyopic eye and fellow healthy eye. After consistency checks, data were transferred to an SPSS spreadsheet (version 25; IBM Corp., Armonk, NY, USA). Qualitative data were presented as frequencies and percentages. If the quantitative data were normally distributed, we estimated the mean and standard deviation. For non-normally distributed data, we estimated the median, interquartile range (IQR), and minimum–maximum values. To compare the data of the amblyopic eye to that of the fellow eye, we correlated them using nonparametric methods to estimate Wilcoxon signed-rank *Z* and *P* values. To compare the values of the amblyopic eye by the time of OCTA, we used a non-parametric method to calculate the Friedman *P* value. To compare the refraction-adjusted change in RVD at the macula in eyes with amblyopia and in the fellow eye to changes in BCVA, we used bivariate correlation parameters in SPSS to estimate the Spearman correlation coefficient and *P-*value. We used a linear regression model of the parametric method using the step-out method to study the interaction of the dependent variables on the correlation with the outcome variable. Statistical significance was defined as a two-sided *P-value* <0.05 was considered statistically significant.

## 3 Results

**Fig. 2:**
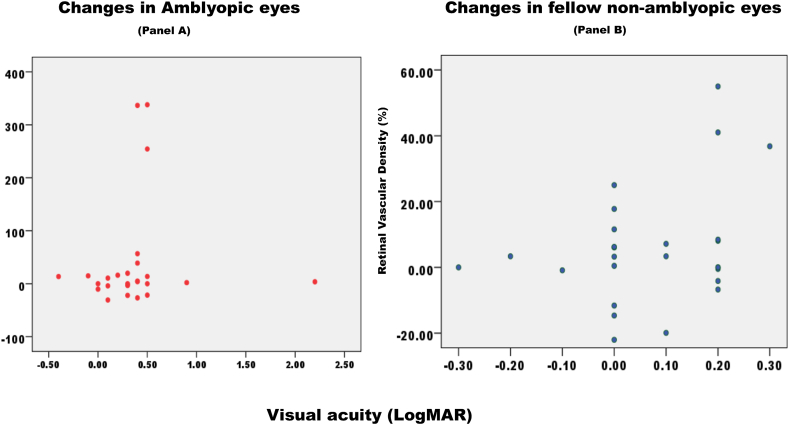
Scatter plot of changes in retinal vascular density and visual acuity.Panel A examines the relationship between changes in best-corrected visual acuity (measured in logMAR) and changes in retinal vascular density (RVD) in amblyopic eyes. The scatter plot depicts a positive correlation, indicating that improvements in visual acuity are accompanied by increases in retinal vascular density. Panel B displays the relationship between alterations in visual acuity (measured in logMAR) and changes in retinal vascular diameter (RVD) in the fellow, non-amblyopic eye. The scatter plot suggests a modest association, with little fluctuation in visual acuity and RVD. The x-axis denotes the change in best-corrected visual acuity (logMAR) at 4th follow up compared to that before amblyopia therapy. The y-axis denotes the percentage change in retinal vascular density at 4th follow up compared to before amblyopia therapy.

Our cohort included 30 amblyopic eyes and 30 fellow eyes from 30 children. Their mean age was 8.7 ± 1.4 years (minimum–maximum; 6–12 years). There were 18 (60 %) boys and 12 (40 %) girls. Unilateral amblyopia occurred in 17 (57 %) right eyes and 13 (43 %) left eyes. Only one child had a history of preterm delivery, while the rest had full-term births. According to the data, strabismus was observed in 19 (63 %) patients with deviation occurring in one eye, while 7 (23 %) patients exhibited strabismus in both eyes. Among the 23 children diagnosed with esotropia, the median deviation (IQR) was 35 (25; 50) prism diopters (PD), and 2 of these children had exotropia with 18 (15; 20) PD. The median degree of strabismus was 30 PD. The management of strabismus included the use of spectacles in 25 (83 %) cases, surgical intervention in 1 (3 %), and botulinum injection in 2 (7 %). Four (13 %) children had high myopia, 3 (10 %) had emmetropia, and 4 (13 %) had mild hyperopia. Nine (30 %) children had moderate hyperopia and 10 (33 %) had severe hyperopia. In addition, 13 (43 %) children had anisometropia. Amblyopia was strabismic in 17 (57 %) of cases, anisometropic in 4 (13 %), and mixed in 9 (30 %).Amblyopia was moderate and severe in 10 and 12 children, respectively. Only 13 (43 %) children had previously undergone occlusion therapy. A comparison of BCVAs in the amblyopic and fellow eyes before and after therapy is shown in Fig. 2. Of the 30 amblyopic children, 24 (80 %) had improved VA at 1st and 2nd follow-up visits, 25 (83 %) on 3rd follow-up, and only 23 (77 %) had improved vision compared to before treatment. The improvement in BCVA in the amblyopic eye at different post-therapy visits was substantial and significantly better than that noted before the start of occlusion therapy (X2 = 57, degrees of freedom = 4, Friedman *P* < 0.001). The improvement in BCVA in the fellow eye at different post-therapy visits was marginal but significant compared to that noted before the start of occlusion therapy (X2 = 16.2, degrees of freedom = 4, Friedman *P* = 0.003). BCVA in the fellow eye was significantly better than that in the amblyopic eye before and at different post-occlusion visits. Refraction in the amblyopic eye was significantly more hyperopic than in the fellow eye (*Z* = 1.79, Wilcoxon signed rank *P* = 0.07).

The total, superior, and inferior to fovea refraction-adjusted RVD in the 3 mm^2^ macular area assessed by OCTA during four visits following occlusion therapy in the amblyopic and fellow eyes are compared in Table 1. The total, superior, and inferior RVD during the first and second OCTAs in the amblyopic eyes were significantly lower than those in the fellow eyes (Wilcoxon signed rank test two-sided *P* < 0.05); however, these parameters were not significantly different between the two eyes at the third and fourth OCTA visits. The total change percentage noted at the fourth OCTA visit compared to the first OCTA visit was significantly higher in the amblyopic eye than in the fellow eyes (two-sided Wilcoxon signed rank test, *P* = 0.05).

**Table 1:**
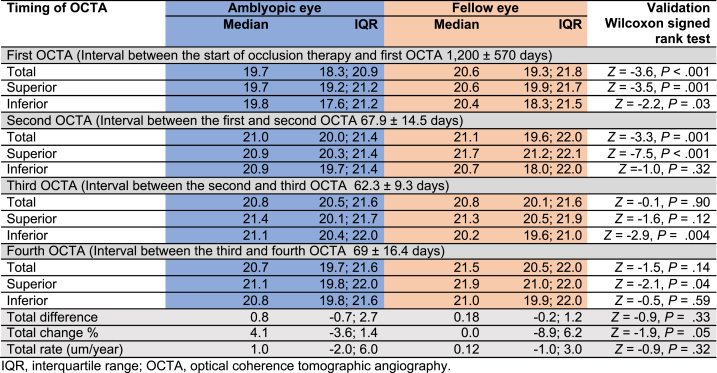
Refraction adjusted retinal vascular density at macula in the amblyopia and fellow eyes.

Changes in refraction-adjusted total RVD at the fourth OCTA visit compared to the first OCTA visit were correlated to changes in BCVA (logMAR) at the fourth OCTA visit compared to the BCVA before amblyopia therapy commenced (Table 2). In amblyopic eyes, the improvement in BCVA correlated significantly and positively with refraction-adjusted total RVD at all visits except the third OCTA visit. In the fellow eye, the change in BCVA was marginal and not significantly correlated with RVD in the second and third OCTAs. Changes in RVD and BCVA at 4th follow-up compared to pre-therapy were compared in amblyopic and fellow non-amblyopic eyes in a scatter plot (Fig. 2).

**Table 2:** Correlation of BCVA and refraction adjusted total RVD at different follow up period post occlusion therapy for unilateral amblyopia in Saudi children.

Amblyopic eye	Change of BCVA (logMAR)		Refraction adjusted total RVD		Spearman correlation coefficient	Spearman two-sided *P* value
	Median	IQR	Median	IQR		
First OCTA visit	0.2	0.0; 0.3	19.7	18.3; 20.9	0.18	0.06
Second OCTA visit	0.3	0.2; 0.45	21.0	20.0; 21.4	0.40	<0.001
Third OCTA visit	0.4	0.3; 0.6	20.8	20.5; 21.6	0.04	0.671
Fourth OCTA visit	0.4	0.3; 0.5	20.7	19.7; 21.6	0.25	0.007
**Fellow eye**						
First OCTA visit	0.0	−0.03; 0.2	20.6	19.3; 21.8	−0.42	<0.001
Second OCTA visit	0.05	0.0; 0.2	21.1	19.6; 22.0	−0.19	0.079
Third OCTA visit	0.05	0.0; 0.2	20.8	20.1; 21.6	−0.09	0.443
Fourth OCTA visit	0.05	0.0; 0.2	21.5	20.5; 22.0	0.29	0.007

BCVA, best corrected visual acuity; IQR, interquartile range; RVD, retinal vascular density.

Linear regression analysis was carried out with refraction adjustment; the outcome variable was the change in BCVA (logMAR), and the dependent variable was the step-out method. The final variables showing a significant correlation included total RVD at the fourth OCTA visit, type of amblyopia, duration of occlusion, and compliance with occlusion therapy (Table 3). Improved vision following patching can be predicted by total RVD, type of amblyopia, duration of occlusion, and compliance with patching in the amblyopic eyes of children (*F* = 11.1, *P* < 0.001).

**Table 3:** Predictors of BCVA post occlusion therapy in amblyopic eye.

Independent variable	Standardized coefficients	95 % confidence interval	*P* value	*T* value
Total RVD at the fourth OCTA visit	0.18	0.005; 0.15	0.036	2.1
Amblyopia type	0.31	0.08; 0.28	<0.001	3.6
Duration of occlusion	−0.42	−0.008; −0.003	<0.001	−4.8
Compliance to occlusion	0.19	0.02; 0.23	0.025	2.3
Constant	–	−2.6; 0.5	−2.8; 0.5	−1.4

BCVA, best corrected visual activity; OCTA, optical coherence tomographic angiography; RVD, retinal vascular density.

## 4 Discussion

### 4.1 Summary of study outcomes

Visual improvement and retinal vascular density changes after occlusion therapy in amblyopic eyes correlated well with our study. The changes in the RVD and BCVA were significantly better in the amblyopic eyes than in the fellow eyes. Refractive status, amblyopia type, duration, and adherence to occlusion therapy influenced BCVA and RVD.

### 4.2 Strength of the present study

Patient selection in the present study was unique and practical because at a tertiary eye center where OCTA facilities usually exist, pediatric ophthalmologists provide care to amblyopic children who are treated. The participants were of school age and not under 5 years old, when amblyopia detection and therapy are usually recommended [20]. The amblyopic eyes responded positively to the monitored occlusion therapy, and visual improvement was observed with improved RVD indices, suggestive of improved macular perfusion. The correlation between visual acuity improvement and OCTA-derived macular perfusion indices has been reported previously [14,21,22]. However, this may be the first study in which serial OCTAs were performed during monitored occlusion therapy for unilateral amblyopia to demonstrate changes over time and the role of patching compliance in such correlations. As suggested by previous researchers, we used SE-adjusted values of RVD to correlate them with changes in BCVA [12].

### 4.3 RVD changes and improvement of vision post amblyopia therapy

A correlation between visual gain and microcirculation after amblyopia therapy was noted in our study. Doğuizi et al. [23] in hyperopic anisometric amblyopic eyes noted a negative correlation between BCVA and foveal density in the superior parafoveal regions of amblyopic eyes. In a study in Hong Kong, the authors found that visual acuity changes were not associated with RVD but were correlated with the foveal avascular zone [11]. In another study, when children responded and did not respond to amblyopia treatment compared to healthy children, vision correlated with outer retinal layer perfusion in amblyopic eyes and hypothesized that light stimulation of the macula could be responsible for RVD improvement and vision [24]. The impact of amblyopia therapy is dependent on compliance with therapy. Visual acuity improvements in the amblyopic eyes of children aged 6–12 years following occlusion therapy for 6–8 months, monitored for the duration of patching per day, and compliance to therapy, in the present study, are promising. Haddad et al. [25] also noted visual recovery in 7-to 12-year-old children with amblyopia, but only in eyes with anisometropic amblyopia. In our study, only four eyes were anisometropic. Visual recovery was also documented in the other types of amblyopic eyes in the present study.

### 4.4 Changes in RVD in amblyopic eyes by therapy

In our study, the RVD in the superficial vascular plexus of the parafoveal area was the index used for the comparison over time of the changes in the amblyopic eye compared to the fellow eye. The changes were marked at the 1st and 2nd follow-ups and then were stable at the 3rd and the 4th follow-up. With only one post-therapy follow-up, Gunzenhauser et al. [14] reported a significant increase in RVD after amblyopia treatment. The outer segment of the retina, which is mainly supplied by the deep vascular plexus and choroidal circulation, improved in thickness one year after treatment and correlated well with visual gain in eyes with anisometropic amblyopia in children [26]. It appears that amblyopia treatment positively affects retinal structures through altered perfusion of this vital area of the retina, resulting in improved visual acuity. However, the changes in different indices in different macular areas and their causal link to visual changes remain an area of further research.

### 4.5 Comparison of outcome in amblyopic eyes to the fellow eye

In our study, we used fellow eyes as a comparison group so that factors such as gender, age, and refractive error (except in eyes with anisometropia) would have minimal variation and thus minimize the bias on the study outcomes. However, the comparison of amblyopic eyes with fellow eyes was less preferable than that of normal healthy children [27]. Interestingly, BCVA in the fellow eye marginally but significantly improved following treatment in the present study. However, the refraction-adjusted percentage of the total RVD change was not significant. Further investigations are required to understand the underlying cause of the association between improved vision and RVD. Whether this represents the natural growth of the macula over time or the indirect effects of amblyopia therapy on the macula of the fellow eye or higher vision center requires further study.

### 4.6 Type of amblyopia and outcomes

Most children had either strabismic or mixed amblyopia. Few patients had anisometropic amblyopia. Therefore, the effect of the type of amblyopia on the correlation between BCVA and RVD changes in our study requires confirmation with larger subgroup samples. Liu et al. noted significant improvements in the superficial retinal capillary plexus density in the macula of anisometropic eyes [16]. Anisometropic amblyopia may respond better than strabismic amblyopia to occlusion therapy, and may affect the association between structural changes and visual gains.

### 4.7 Timing of OCTA after therapy

At the first and second OCTA, with a median duration of 3 years of treatment and 2 months after the first OCTA, respectively, RVD significantly improved. On the third and fourth OCTA scans, it was stable and not different from the RVD in the fellow eyes. Proper timing of evaluation of structural changes seems to be crucial when using OCTA to monitor occlusion therapy.

### 4.8 Compliance of amblyopia therapy and outcomes

In our study, compliance with occlusion therapy was an independent predictor of the correlation between BCVA gain and RVD changes. Furthermore, the correlation between compliance with prescribed occlusion times, as assessed by orthoptists, and improved visual outcomes underscores the importance of adherence to the treatment protocols. Notably, the gradation of the daily occlusion time based on the severity of amblyopia, as used in our study, aligns with the personalized treatment approach recommended by Jia et al. (2023) [28]. Compliance with the prescribed occlusion regimen and alterations in the duration per day are crucial for achieving the desired outcomes. Parents and caregivers at schools should be counseled to ensure better compliance of amblyopic children with occlusion of the non-amblyopic eye, as advised by the therapist. Our study's extension of the period of occlusion to one year offers a unique perspective on the long-term vascular changes associated with patching therapy, contributing to ongoing discussions on optimal treatment durations highlighted in recent publications [29].

### 4.9 RVD by regions and outcomes

We used the superior and inferior parafoveal regions of the macula to note the RVD changes after occlusion therapy, in addition to the percentage changes in the RVD in the total macular area. In amblyopic eyes, the RVD increase was similar in the superior and inferior parafoveal areas and equally correlated with changes in BCVA at the time of the first and second OCTAs; however, RVD improvement was sustained in the superior area but not in the inferior area of the amblyopic eyes on the fourth OCTA visit. Such variations in RVD were also observed by Liu et al., in which nine areas of the macula were studied for RVD changes in amblyopic eyes [22]. Our research findings are consistent with the recent literature and emerging trends in the field of amblyopia therapy, particularly with regard to retinal vascular density. AttaAllah et al. [30] demonstrated that following successful amblyopia treatment, patients exhibited enhanced best-corrected visual acuity and improved macular perfusion, particularly in the superficial and deep vascular density, as well as choriocapillaris foveal RVD.

## 5 Limitations of the present study

Our study had some limitations. Because of the small sample size, internal comparisons of the subsamples showed trends and need to be interpreted with caution. Foveal avascular zone diameter was not used as an OCTA index to correlate with visual recovery in our study. The axial length (AL) was not documented. Since three-quarters of the participants had hyperopia in their amblyopic eye, AL is a more influential factor on macular structural changes in high-myopic eyes [31], is less likely to have affected our study outcomes, and refraction-adjusted RVD enabled us to minimize axial length-related bias in our study. Our study outcomes were based on SE-adjusted RVD parameters. Some studies have not included such adjusted parameters [14,15]. Hence, comparing our study outcomes to these studies should be performed with caution.

### 5.1 Clinical implications of the present study

Based on our study outcomes, pediatric ophthalmologists have suggested using OCTA as a tool to monitor perfusion changes as a proxy indicator of physiological changes in the macula and predict visual gain after supervised amblyopia therapy. They should also emphasize on parents and teachers the role of compliance in therapy to achieve desired outcomes.

## 6 Conclusions

As amblyopia affects children, disability-adjusted life years lost due to this ailment pose a high disease burden and require interventions with long-term success. Understanding the hemodynamic characteristics of the macula and the impact of occlusion therapy on amblyopia is vital for pediatric ophthalmologists and researchers to understand the link between structural and visual functional changes in older children with amblyopia. The use of new technologies to understand the pathophysiology and impact of interventions will improve health professionals' confidence and parents’ trust in these treatments, which are often challenging for children and their caregivers.

## Ethical statement

The study was authorized by the Ethics and Research Committee of King Khaled Eye Specialist Hospital, and the approval number for this study was 20139-P. Moreover, written informed consent was obtained from the legal guardians or parents of all participating minors. The research followed the principles outlined in the Declaration of Helsinki. All procedures were carried out in accordance with the ethical guidelines established by the institutional and national research committee, as well as the 1964 Helsinki declaration and its subsequent amendments or equivalent ethical standards.

## Data availability statement

The datasets that were either generated or analyzed during the current study are accessible from the corresponding author upon request. For such requests, it is necessary to include a comprehensive justification that will be assessed by the research team. Approval from the institution and adherence to ethical standards will be followed while providing the data.

## CRediT authorship contribution statement

**Gorka Sesma:** Writing – review & editing, Writing – original draft, Supervision, Software, Methodology, Investigation, Formal analysis, Data curation, Conceptualization. **Tasnim Almairi:** Writing – review & editing, Writing – original draft, Visualization, Project administration, Methodology, Investigation, Formal analysis, Data curation, Conceptualization. **Heba Khashoggi:** Visualization, Resources, Project administration, Investigation, Data curation. **Fahad Aljohar:** Visualization, Software, Resources, Project administration, Data curation. **Rajiv Khandekar:** Writing – original draft, Supervision, Methodology, Investigation, Formal analysis, Data curation, Conceptualization. **Abdulaziz Awad:** Writing – review & editing, Writing – original draft, Validation, Supervision, Conceptualization. Gorka Sesma and Tasnim Almairi contributed equally to this work and should be considered co-first authors.

## Declaration of competing interest

The authors declare that they have no known competing financial interests or personal relationships that could have appeared to influence the work reported in this paper.
